# Characterization of *Sphingobacterium* sp. Ab3 Lipase and Its Coexpression with LEA Peptides

**DOI:** 10.1128/spectrum.01422-21

**Published:** 2022-10-31

**Authors:** You Kiat Ng, Shinya Ikeno, Ammar Khazaal Kadhim Almansoori, Ibrahim Muhammad, Rashidah Abdul Rahim

**Affiliations:** a School of Biological Sciences, Universiti Sains Malaysia, Penang, Malaysia; b Department of Biological Functions and Engineering, Graduate School of Life Science and System Engineering, Kyushu Institute of Technology, Kitakyushu, Japan; c Department of Science Lab. Technology, Ramat Polytechnic Maiduguri, Maiduguri, Nigeria; Swansea University

**Keywords:** *Sphingobacterium* sp., lipase, microbial biotechnology, protein expression, characterization

## Abstract

*Sphingobacterium* sp. is a yellowish Gram-negative bacterium that is usually characterized by high concentrations of sphingophospholipids as lipid components. As microbial enzymes have been in high demand in industrial fields in the past few decades, this study hopes to provide significant information on lipase activities of *Sphingobacterium* sp., since limited studies have been conducted on the *Sphingobacterium* sp. lipase. A microbe from one collected Artic soil sample, ARC4, was identified as psychrotolerant *Sphingobacterium* sp., and it could grow in temperatures ranging from 0°C to 24°C. The expression of *Sphingobacterium* sp. lipase was successfully performed through an efficient approach of utilizing mutated group 3 late embryogenesis abundant (G3LEA) proteins developed from Polypedilum vanderplanki. Purified enzyme was characterized using a few parameters, such as temperature, pH, metal ion cofactors, organic solvents, and detergents. The expressed enzyme is reported to be cold adapted and has the capability to work efficiently under neutral pH (pH 5.0 to 7.0), cofactors like Na^+^ ion, and the water-like solvent methanol. Addition of nonionic detergents greatly enhanced the activity of purified enzyme.

**IMPORTANCE** The mechanism of action of LEA proteins has remained unknown to many; in this study we reveal their presence and improved protein expression due to the molecular shielding effect reported by others. This paper should be regarded as a useful example of using such proteins to influence an existing expression system to produce difficult-to-express proteins.

## INTRODUCTION

In recent decades, the role of enzymes as catalysts has been essential to industry, especially the food industry. Enzymes are industrially important because of their high specificity and capability of being active under mild conditions, which ensure low waste production and treatment costs (1–3). In most industries, microbial enzymes are the most preferable choices, rather than animal or plant enzymes, due to the absence of seasonal fluctuations (1). Aside from catalyzing a broad range of reactions, the high stability of microbial enzymes under varied conditions allows improved quality control in terms of industrial production and product preservation (1, 4, 5). Unlike animal and plant enzymes, microbial enzymes can be genetically modified easily and produced in large quantities consistently at minimal costs (1, 4, 6). A well-rounded utilization of microbial enzymes often allows most industrial processes and productions to be inexpensive, convenient, and environmentally safer (1, 4, 6). With these enzymes as catalysts, industrial processes and productions can be inexpensive, more convenient, and safer (1, 4, 6). Lipase is a hydrolase that is commonly used for transesterification in various industrial applications, such as food processing and deinking processes (1, 5, 7).

While providing these advantages, common enzymes are inapplicable in some industrial processes that are conducted under extreme conditions in terms of pH, temperature, and salinity (8, 9). Under these extreme conditions, common enzymes would experience aggregation, precipitation, and denaturation, in which their enzymatic activities would be drastically reduced (8, 10). Enzymes from extremophiles, known as extremozymes, are the most widely studied, as these enzymes are capable of catalyzing similar enzymatic reactions under extreme conditions which could greatly incapacitate their nonextreme counterparts (8–10).

Recently, much attention has shifted toward cold-adapted lipases from psychrophiles as potential enzymes to be applied in cold industrial processes, such as cold washing and skin dehairing processes (11, 12). At low temperature, water molecules become more ordered, and this reduces the interactions with common enzymes, pushing these enzymes toward the unfolded and denatured state (8). While adapting to such an environment, cold-adapted lipases are observed to have a flexible structure, which allows them to hold onto water molecules tightly, sustaining their lipolytic activity with minimal energy requirement (8, 9). Such unique characteristics of cold-adapted lipases have become a main interest, as these enzymes offer similar enzymatic activities as their nonextreme counterparts while providing good product quality preservation by limiting the high temperature requirement (9). Candida antarctica lipase A (CAL-A) and CAL-B are the most well-known cold-adapted lipases of C. antarctica from Antarctica, which have been mostly utilized in the industrial fields (13).

Late embryogenesis abundant (LEA) proteins are a group of hydrophilic proteins that are induced for anhydrobiosis in both plants and animals. LEA proteins are categorized into six families based on the similarities of amino acid sequences and unique conserved motifs (14). Although a variety of LEA proteins exist, all LEA proteins share common traits such as high hydrophilicity, high content of Gly, and small amino acids like Ala and Ser (14). Group 3 LEA (G3LEA) proteins are generally characterized by a repeating motif of 11 amino acids (14). Most of the G3LEA proteins have been reported to serve a protective role for plants and animals during the onset of water stress conditions (15–21). Recently, a novel approach of involving G3LEA protein in coexpression with target protein within its host, Escherichia coli, enhanced the protein solubility drastically, leading to better production of functional protein (22–24). Unlike others, the method is simple and efficient by only utilizing the conserved 11 amino acid residues of G3LEA protein (23). Improvement of this method is also notable as it only manipulates hydrophobic, charged amino acids among 11 amino acid residues of G3LEA protein via point mutations (24). Since the coexpressed peptide is small, the impact toward the functional protein is insignificant, and this allows simple, convenient protein purification (23). We hypothesized that, by utilizing the advantage of the properties of G3LEA proteins, such as LEA-I and LEA-K, in molecular shielding, the nature of lipase as highly toxic toward its host, Escherichia coli, might be overcome, leading to efficient protein expression.

The genus *Sphingobacterium* in 1983 was distinguished from *Flavobacterium* sp. by its high concentrations of sphingophospholipids (25). *Sphingobacterium* is the type genus of the family *Sphingobacteriaceae* in the phylum *Bacteroidetes* (26). *Sphingobacterium* spp. are ubiquitous in nature, being found in soil (27, 28) and compost or activated sludge (29, 30), and also sometimes from clinical specimens (31). Most *Sphingobacterium* species are Gram-negative, nonfermenting, non-spore-forming, rod-shaped bacteria, and there are 24 species of *Sphingobacterium* that are currently recognized (32). A few species are associated with human infections, except for Sphingobacterium spiritivorum and Sphingobacterium multivorum, which were isolated from bloodstream infections, respiratory infections, cellulitis, and end-stage kidney disease (31). Decades ago, only a few authors had reported that *Sphingobacterium* species possessed high lipolytic activities, but these findings were supported by relatively little evidence (33, 34). To relieve such a situation, those authors suggested that studies were required to further understand the properties of *Sphingobacterium* sp. lipase for possible future industrial applications (34). In this study, *Sphingobacterium* sp. was one of the microbes that were previously discovered and isolated from the Artic soil sample ARC4. The objective of this study was to express and characterize the lipase gene of *Sphingobacterium* sp. obtained from an Artic soil sample.

## RESULTS AND DISCUSSION

### Screening of the lipase-producing strain and PCR amplification.

**FIG 1:**
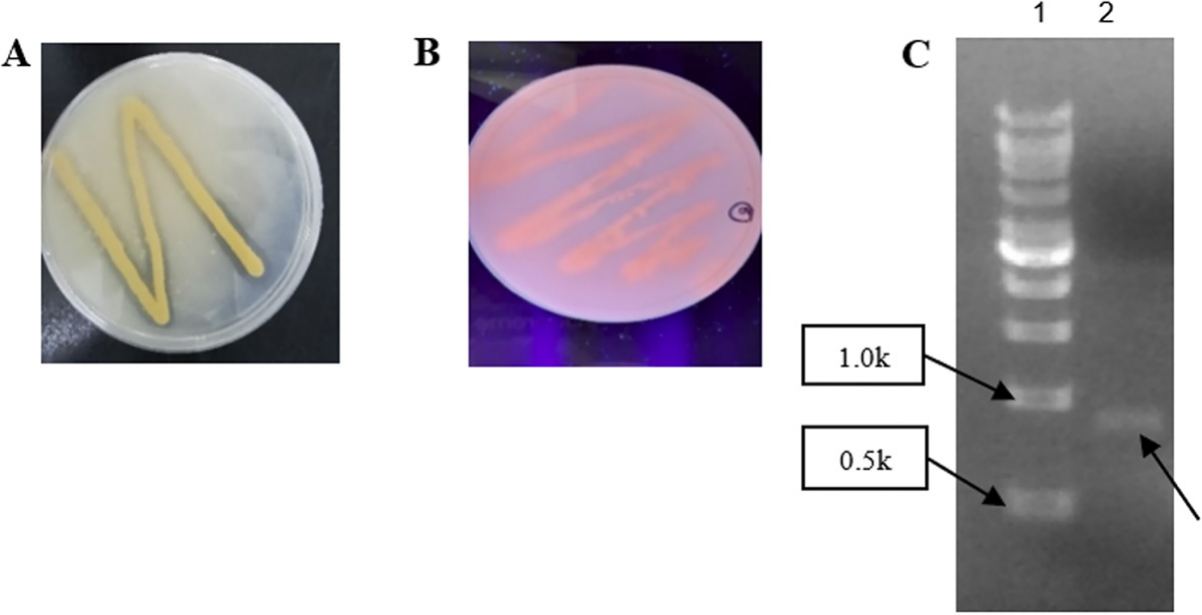
(A) Tributyrin test. Presence of a clear halo zone indicated the presence of esterase or lipase from *Sphingobacterium* sp. (B) Rhodamine B test for further screening of the lipase-producing strain. Formation of orange fluorescence indicated only the presence of lipase activity from *Sphingobacterium* sp. (C) PCR amplification of the Ab3 gene. Lane 1, Vivantis 1-kb DNA ladder; lane 2, Ab3 gene product with a size of ~1.0 kb.

In this study, qualitative tests on tributyrin and rhodamine B agar plates were used for detection of any lipase activity present from the bacterial isolate. Tributyrin agar is frequently used for detection of extracellular lipase activity of an organism (35). In this experiment, the medium was prepared using Luria-Bertani agar and 1% triglyceride tributyrin. As tributyrin is dispersed in the agar, the formation of an emulsion causes the medium to become opaque, which is necessary for observation of any lipase activities (36); using triglyceride tributyrin as a lipase substrate, any lipase-producing organism would show a clear zone of lipolysis, which is shown in Fig. 1A as a positive result. However, false-positive results could occur, considering the presence of esterase that could hydrolyze tributyrin as well (36, 37).

At present, the use of rhodamine B as the fluorescent dye for screening lipolytic organisms is widely practiced, as rhodamine B does not affect the growth of the microorganisms (38). Thus, rhodamine B-olive oil agar was suitable to serve as a reinforcement to support the results from the tributyrin test (35). During the process, rhodamine B tends to form complexes with free fatty acids produced only by extracellular lipase released by the specimen, causing formation of orange fluorescent under UV irradiation of 340 nm (38). As shown in Fig. 1B, orange fluorescence formed among *Sphingobacterium* sp. colonies after 48 h of incubation at 24°C, indicating only the presence of true lipase. Hence, the bacterial isolate was concluded to be a lipase-producing strain.

Through PCR amplification via Ab3F and Ab3R, amplified PCR fragments with a size of ~1.0 kb were obtained, and the Ab3 gene wa successfully produced (Fig. 1C, lane 2).

### Expression of recombinant protein via pCold-I plasmid.

**FIG 2:**
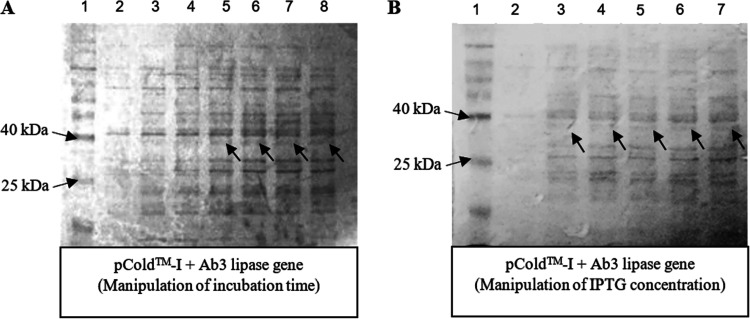
(A) Expression level via pCold-I plasmid at different incubation times at 15°C and 0.1 mM IPTG concentration. Lane 1, Vivantis protein ladder (10 to 175 kDa); lane 2, control (no incubation); lanes 3 to 8, expression levels of Ab3 lipase at different incubation times (4, 8, 12, 24, 48, and 72 h, respectively). (B) Expression levels via pCold-I plasmid using different IPTG concentrations at 15°C. Lane 1, Vivantis protein ladder (10 to 175 kDa); lane 2, control (no IPTG); lanes 3 to 7, expression levels of Ab3 lipase at different IPTG concentrations (0.2, 0.4, 0.6, 0.8, and 1.0 M IPTG, respectively).

After overnight incubation, only a faded desired protein band (~36 kDa) was observed at the end of recombinant protein expression via pCold-I. The cause of the phenomenon was suspected to be the lack of suitable protein expression conditions. Since protein expression was conducted at 15°C, the protein expression was expected to be slower, indicating a longer incubation time may be required. As shown in Fig. 2A, the protein expression was observed to be halted after 24 h of incubation, as the intensity level of protein bands remained uniform after 72 h of incubation.

As longer incubation did not solve the poor protein expression, further optimization was then conducted by adjusting the isopropyl-β-d-thiogalactopyranoside (IPTG) concentrations. Regulation of inducer concentration was crucial in improving the protein expression in E. coli (39, 40). Presence of mutation in lac permease (*lacY*) allowed IPTG to uniformly penetrate the cells, resulting in a concentration-dependent and homogeneous level of induction (39). Inappropriate IPTG concentrations could either lead to insufficient induction of protein expression or terminate the protein expression due to a toxic effect toward E. coli (40, 41). According to several studies, the appropriate IPTG concentrations a reported to be between 0.1 and 1.0 mM to prevent any toxic effect toward the growth of E. coli (39, 42). From Fig. 2B, the intensity of the desired protein band was observed to remain faded and uniform in the presence of different IPTG concentrations (0.2 to 1.0 mM) after 24 h of incubation at 15°C, indicating the expression level of Ab3 protein was either unaffected or improved.

Throughout the protein expression, the expression level of Ab3 protein remained constantly low, as shown by the SDS-PAGE results (Fig. 2A and B). It was highly suspected that the low expression level of Ab3 protein was correlated to the nature of the Ab3 protein itself at low temperature. The psychrophilic enzymes are crucial as one of the low-temperature adaptation mechanisms of the psychrophiles, indicating the importance of folding and refolding at low temperature (43). The structure of the psychrophilic enzymes was highly flexible but with low stability, which led to its structure being easily denatured when exposed to heat (44, 45). During expression and purification, the expressed proteins are required to be properly folded and structurally stable (46). Proteins with low structural stability experience a high risk of degradation, causing insufficient proteins to be produced (46). Many proteins are expressed with tags or fusion partners to prevent proteolytic degradation and increase protein stability by promoting correct protein folding (47–49).

This also indicated that there could be other factors inhibiting the protein expression, such as the lack of suitable molecular chaperones and an incompatible host. The solubility of the protein produced by Escherichia coli is not always guaranteed, as the generated proteins tend to degrade or aggregate, leading to formation of inclusion bodies, which affect the function of the protein (50, 51). Molecular chaperones are ubiquitous proteins that assist newly synthesized polypeptides and denatured proteins to achieve the native conformation (43, 52). Coexpression with a suitable molecular chaperone has also been reported to prevent protein aggregation and enhance protein stability (46).

However, there were many optimizations available to be performed to improve the expression of Ab3 protein using the pCold-I vector by investigating these potential factors to overcome. By considering times and efforts, alternate strategies were devised to conduct the protein expression of the Ab3 lipase gene.

### Coexpression of recombinant protein and LEA peptides.

G3LEA proteins such as LEA-I (23) and LEA-K peptides (24) were used for coexpression with Ab3 lipase to improve Ab3 lipase protein expression. A few reports have demonstrated that the presence of G3LEA proteins can provide efficient protein expression due to their function in “molecular shielding” (22–24).

The specific vector, pCold-I, may not be considered suitable for coexpression with LEA peptides, as the pCold-I vector contains one multiple-cloning site whereby only one desired gene can be ligated. Besides, the bridge nucleotide sequence may be required to combine the Ab3 gene with LEA peptide sequence for fitting into the pCold-I vector. However, this process may be tedious and inefficient, as this method may bring some complications, such as a potential frameshift between the nucleotide sequences and improper restriction digestions during the combination. Thus, pRSFDuet-I was chosen for such a task instead of pCold-I, as pRSFDuet-1 contains two separate multiple-cloning sites in which the Ab3 gene and LEA peptide sequence could be ligated toward each site. By using pRSFDuet-I, the cloning process required less effort and was more time-saving.

**FIG 3:**
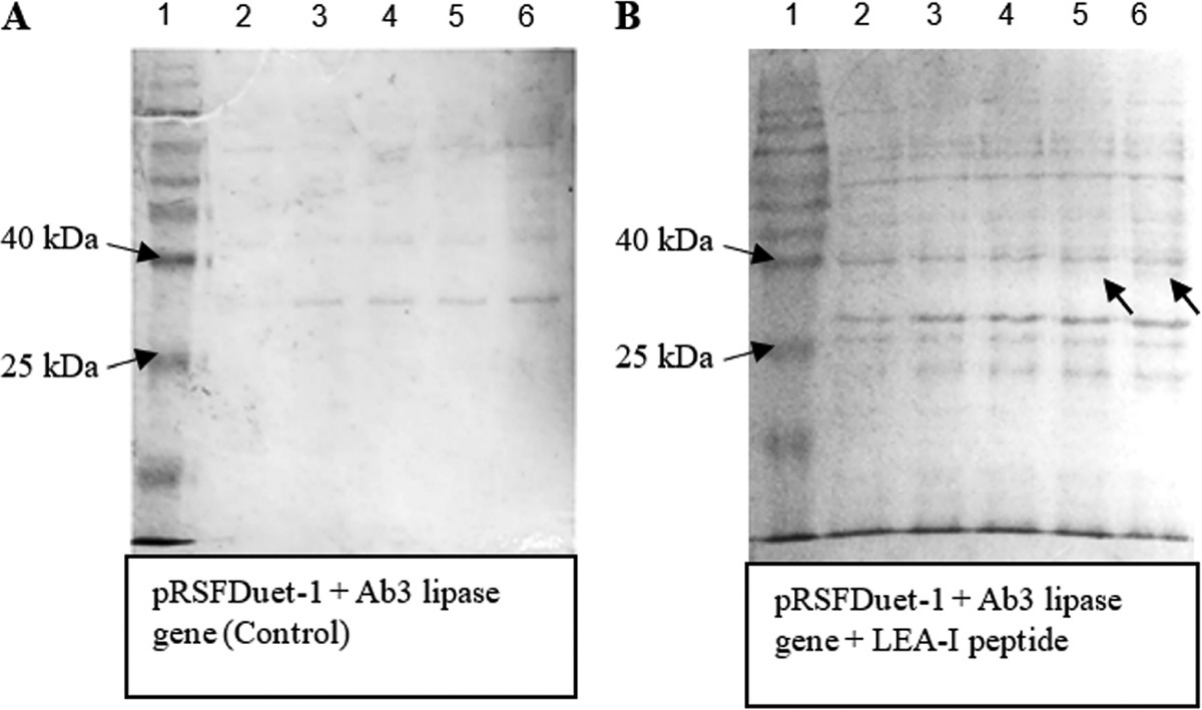
(A) Protein expression without any LEA peptides at 15°C and 0.1 mM IPTG concentration. Lane 1, Vivantis protein ladder (10 to 175 kDa); lanes 2 to 6, expression levels from 0 to 24 h. (B) Coexpression of Ab3 gene with LEA-I peptide at 15°C and 0.1 mM IPTG concentration. Lane 1, Vivantis protein ladder (10 to 175 kDa); lanes 2 to 6, expression levels from 0 to 24 h. Faded protein bands were only seen at 12 and 24 h.

**FIG 4:**
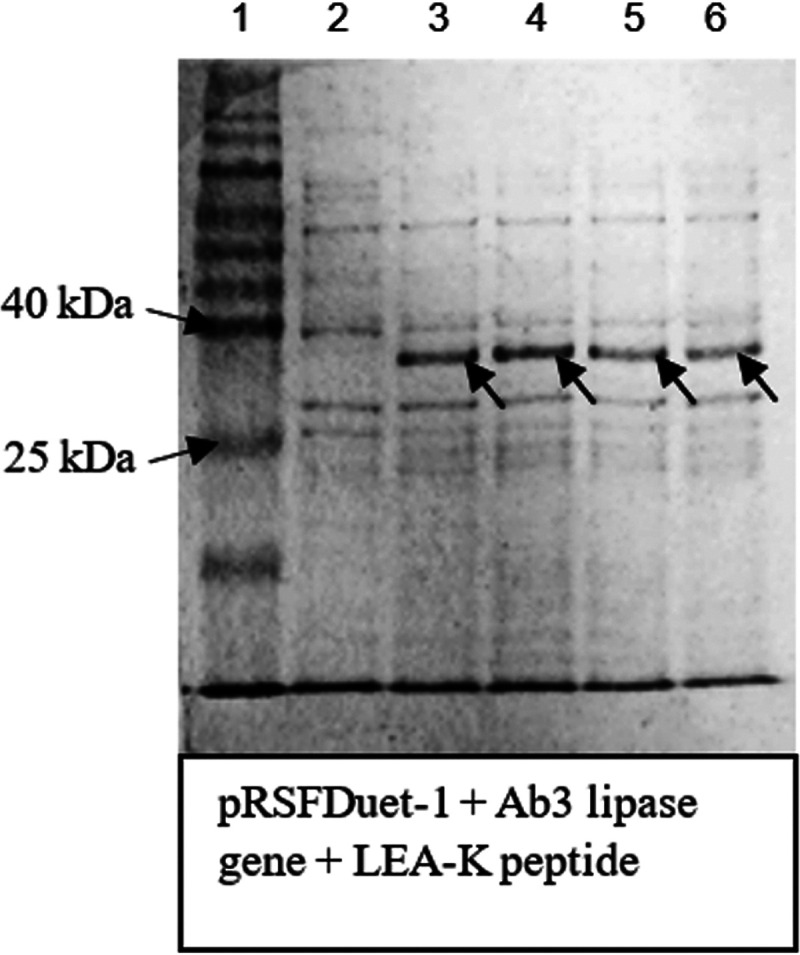
Coexpression of Ab3 gene with LEA-K peptide at 15°C and 0.1 mM IPTG concentration. Lane 1, Vivantis protein ladder (10 to 175 kDa); lanes 2 to 6, expression levels from 0 to 24 h. Thick protein bands were seen throughout 24 h, which indicated successful protein expression.

In Fig. 3A, without LEA peptides, normal Ab3 protein expression was shown to be poor due to the high toxicity of Ab3 protein. As the study proceeded, coexpression of Ab3 lipase via LEA peptides was successful, as shown in Fig. 3B and Fig. 4. However, LEA-I peptide demonstrated poor protein expression compared to its counterpart, LEA-K peptide. LEA-K peptide is more positively charged and bigger than the LEA-I peptide (24). LEA peptide, with its higher number of polar amino acid residues and larger size, may help to stabilize the structure of macromolecule membranes such as proteins, while forming a physical barrier between biomolecules and membranes, preventing their contact with each other (23, 24). In that sense, LEA-K peptide was proven to be a suitable choice for Ab3 lipase protein expression.

**FIG 5:**
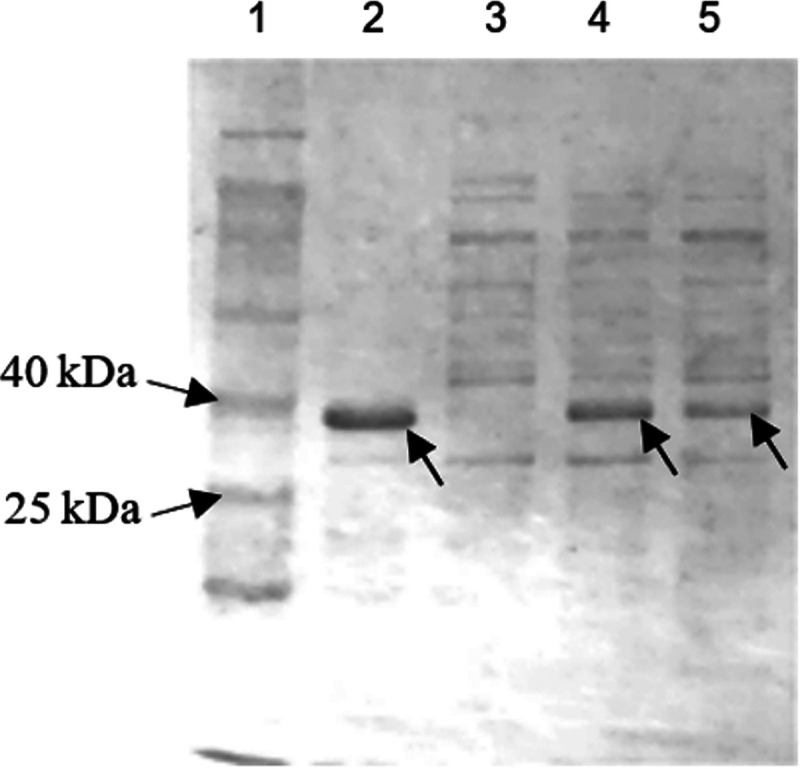
Optimization of protein expression. Lane 1, Vivantis protein ladder (10 to 175 kDa); lanes 2 and 3, Ab3 lipase was initially present as an inclusion body (insoluble form) after sonication; lanes 4 and 5, both insoluble and soluble proteins, respectively, showed an increase in the solubility of Ab3 lipase after optimizations made toward certain protein expression conditions, such as pH, temperature, and addition of glycerol.

Figure 5 shows that the Ab3 lipase protein was mostly present as inclusion bodies after initial protein expression via LEA-K peptide. This indicated that some optimizations were required to increase protein solubility. First, the incubation temperature was reduced to 24°C to reduce the rate of protein aggregation. Protein aggregations are generally favored at high temperature because most hydrophobic interactions in proteins are temperature dependent (39, 50, 53). By lowering the expression temperature, the protein folding rate became slower, promoting the correct folding of the protein (54–56).

**FIG 6:**
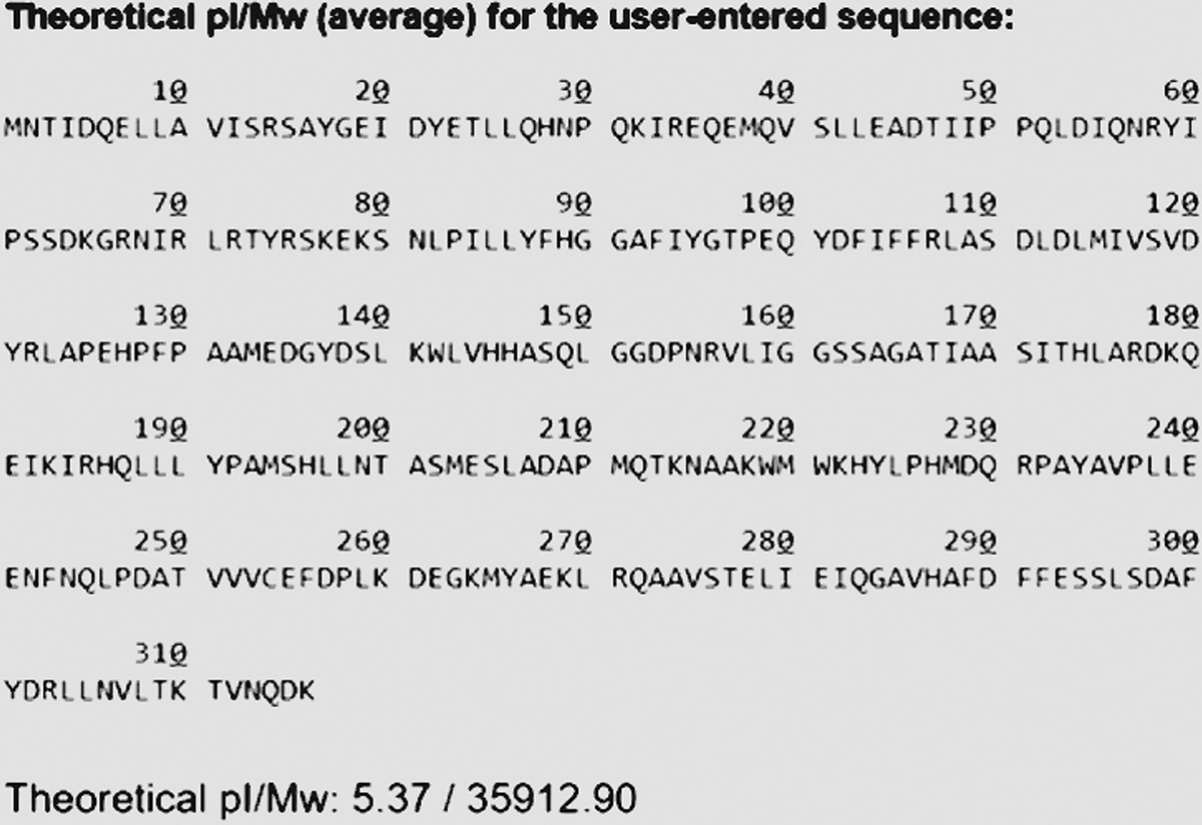
Analysis of the lipase gene via Expasy. The estimated protein molecular weight of the lipase was 35,912.9 Da, or 36 kDa.

Some optimizations were made toward the pH of the storage buffer used, sodium phosphate buffer. A protein has the least solubility at the isoelectric point (pI), whereby occurrence of accumulation in protein-protein interactions leads to protein aggregations due to fewer interactions between water and the protein molecules when the electrostatic forces of the protein molecules are at a minimum (57–59). From Fig. 6, the theoretical isoelectric point of Ab3 protein was 5.3. Hence, the pH of the sodium phosphate buffer was adjusted to pH 7.0 to increase protein solubility.

The protein solubility was further increased by addition of glycerol in the storage buffer. Glycerol is well known as a mild protein stabilizer, as this polyol osmolyte may form certain intermediates that are stabilized by hydrophobic interactions of the protein molecule, inhibiting any intermolecular protein aggregations (60–62). Together with LEA peptides, the presence of glycerol also provided an additional layer of protection for Ab3 proteins from any thermal denaturation. This led to better preservation of Ab3 proteins by using the prepared storage buffer before conducting protein purification. By applying all the optimizations mentioned, the highly soluble Ab3 protein was obtained as the product of protein expression (Fig. 5, lanes 4 and 5) after sonication.

### Purification of expressed protein.

**FIG 7:**
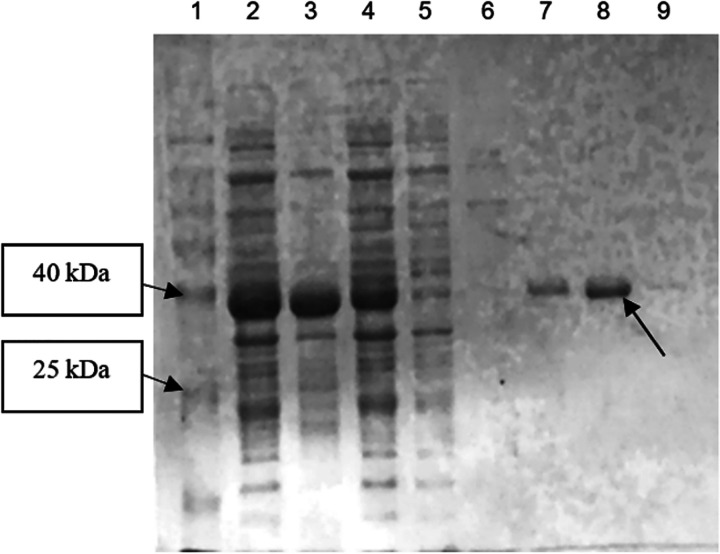
Overall SDS-PAGE result for His-tag purification. Lane 1, Vivantis chromatin prestained protein ladder (10 to 175 kDa); lane 2, total protein before sonication; lanes 3 and 4, insoluble and soluble proteins (after cell lysis), respectively, where the protein was determined to be partially soluble; lane 5, flowthrough collected from the beginning of protein purification; lanes 6 to 9, enzyme purification conducted by using various imidazole concentrations (10, 20, 40, and 60 mM, respectively). In the end, purified Ab3 lipase was obtained.

As shown in Table 1, Ab3 lipase was purified with a 92.7% yield by 34-fold purification. Based on the observed SDS-PAGE result (Fig. 7), a single band was found in the presence of 40 mM imidazole in the HisTalon metal chromatography (Fig. 7, lane 8), which indicated that the expressed protein was successfully purified.

**TABLE 1:** Analysis of protein purification method^*a*^

Purification method	Total act (U)	Total protein (μg)	Sp act (U/μg)	Purification (fold)	Yield (%)
Crude protein	164.8	2680.0	0.0615	1	100.0
HisTalon chromatography	153.6	72.6	2.1157	34.4	92.7

a Total activity was calculated after determination of lipase activity, which was conducted at 15°C for 30 min. Total protein was calculated after estimation of protein concentration in a Bradford assay.

### Biochemical characterization.

**FIG 8:**
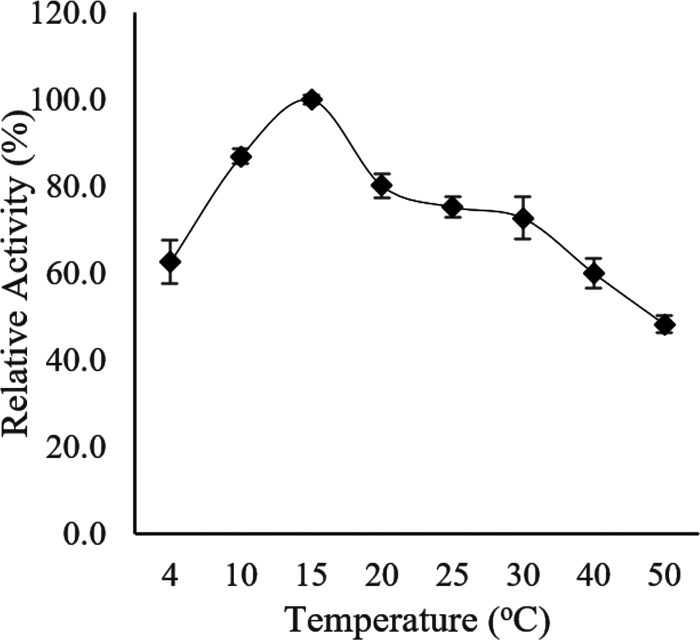
Optimal temperature for activity of Ab3 lipase. Purified Ab3 lipase was incubated at various temperatures during a standard lipase assay protocol. Specific activity of Ab3 lipase (88.5 ± 1.0 U/μg) at 15°C was used as control (100%). All data for both results are expressed as means ± standard deviations of the lipase assay performed in triplicates.

**(i) Effect of temperature on enzyme activity and thermostability.** To determine the optimal temperature for Ab3 lipase activity, the enzyme assay was conducted over a temperature range between 4°C and 50°C (Fig. 8). An initial study showed that Ab3 lipase activity was first observed to slowly increase when the temperature increased toward 10°C, until the activity reached its peak, which was 77.0 ± 1.4 U/μg. The Ab3 lipase activity dropped drastically upon further temperature elevation from 20°C to 50°C.

As the temperature range was narrowed to between 10°C and 30°C, a similar pattern as from the initial finding was observed from 4°C to 10°C. However, the activity further increased until it reached the maximal peak at 15°C, which was 88.5 ± 1.0 U/μg. Afterwards, the Ab3 lipase activity began to decline sharply, up to 30% at the temperature range between 20°C and 30°C. This showed that the optimal temperature of Ab3 lipase activity was at 15°C instead of 10°C. Thus, Ab3 lipase activity at 15°C was used as a control value (100%) for comparative studies in later biochemical characterizations.

**FIG 9:**
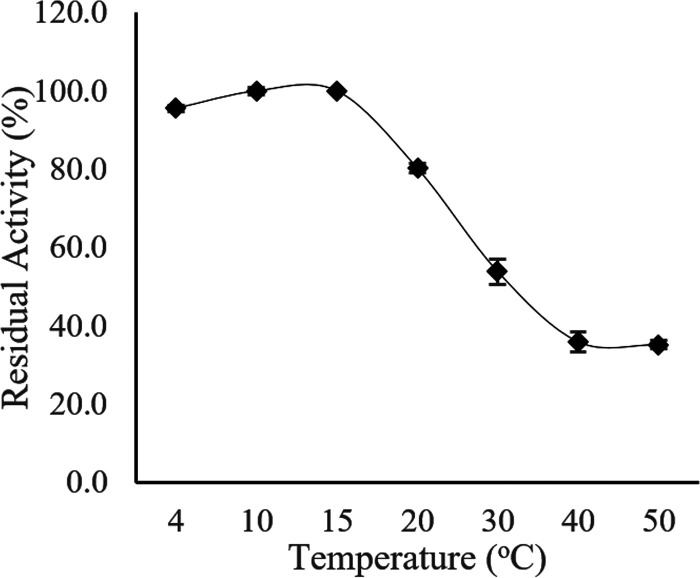
Temperature stability for the activity of Ab3 lipase. Purified Ab3 lipase was exposed directly to various temperatures through preincubation before a standard lipase assay protocol. Specific activity of Ab3 lipase at 15°C (87.4 ± 0.1 U/μg) was used as control (100%). Results are expressed as means ± standard deviations of the lipase assay performed in triplicates.

Thermostability of Ab3 lipase was then studied by preincubating the Ab3 lipase in 0.1M sodium phosphate buffer (pH 7.4) at different temperatures prior to a standard enzyme assay. As shown in Fig. 9, Ab3 lipase retained most of its enzymatic stability from 4°C to 15°C and exhibited its maximal lipase activity. However, as the temperature increased from 20°C to 50°C, a sharp decrease in the stability of Ab3 lipase was notably observed, losing most of its enzymatic activity, up to 70%. This indicated that Ab3 lipase was thermolabile, a behavior shown in most cold-adapted enzymes which possess high vulnerability toward high temperature. Yet, Ab3 lipase possibly possesses moderate thermolabile behavior, as Ab3 lipase was able to retain 80% of its enzymatic activity at 20°C (Fig. 8).

In comparison with Fig. 8 and 9, the study showed that Ab3 lipase was shown to be a cold-adapted enzyme which exhibited high enzymatic activity at a temperature not more than 15°C with moderate thermostability properties.

**FIG 10:**
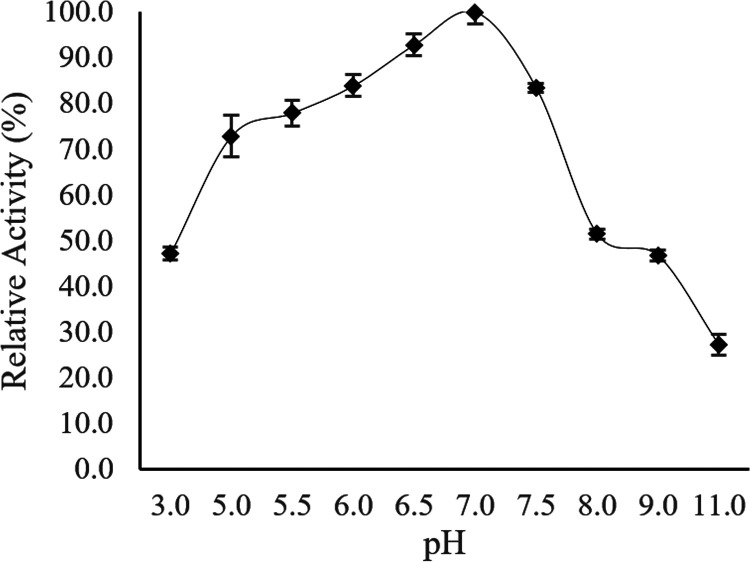
Effect of pH toward the activity of Ab3 lipase. Purified Ab3 lipase was incubated with various buffers with different pHs during a standard lipase assay protocol. Specific activity of Ab3 lipase at pH 7.0 and 15°C (83.9 ± 1.7 U/μg) was used as control (100%). Results are expressed as means ± standard deviations of the lipase assay performed in triplicates.

**(ii) Effect of pH on enzyme activity and stability.** The optimal pH of Ab3 lipase activity was determined by using 1.0% olive oil as a substrate at various pH levels in various buffers at 15°C. As shown in Fig. 10, the initial finding indicated that the purified Ab3 lipase exhibited its highest activity at an optimum pH of 7.0 in 0.1 M sodium phosphate buffer at 15°C. The high activity of Ab3 lipase was notably detected at a pH range between 5.0 and 7.0, as the lipase activity in these ranges was found to be more than 70%. As the pH range narrowed between 5.0 and 8.0, the Ab3 lipase activity was observed to be steadily increasing toward its highest activity, which was 83.9 ± 1.7 U/μg as the pH shifted toward pH 7.0. The optimal pH for the Ab3 lipase activity was further determined to be at pH 7.0 and this was used as for the control value (100%) for comparative studies in later biochemical characterizations.

**FIG 11:**
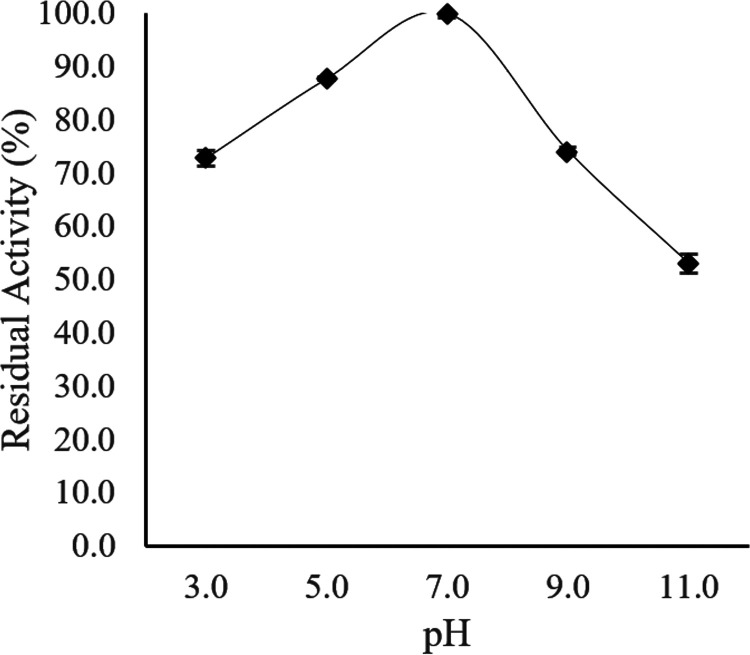
pH stability for the activity of Ab3 lipase. Purified Ab3 lipase was exposed directly to various buffers with different pHs through preincubation for 30 min before a standard lipase assay protocol. Specific activity of Ab3 lipase at pH 7.0 and 15°C (87.4 ± 0.7 U/μg ) was used as a control (100%). Results are expressed as means ± standard deviations of the lipase assay performed in triplicates.

The pH stability of Ab3 lipase was then studied by preincubating the Ab3 lipase in different buffers at various pHs at 15°C for 30 min prior to a standard enzyme assay. Figure 11 shows that Ab3 lipase was capable of retain more than 80% of its enzymatic stability over a pH range between 5.0 and 7.0, similar to the results shown in Fig. 10. Simultaneously, poor stability was mostly observed at other pH ranges. However, Ab3 lipase managed to retain more than 50% of its enzymatic activity under extreme pH conditions (pH 3.0 and 11.0), which explained the presence of enzymatic activity at the pH ranges shown in Fig. 10. Despite having low enzymatic activity under extreme pH conditions, the Ab3 lipase possibly possessed some extent of tolerance under extreme pH conditions.

In comparison with Fig. 10 and 11, the study showed that Ab3 lipase was a cold-adapted enzyme which preferred to function at pH 7.0 while possessing high tolerance toward extreme pH environments (pH 3.0 and 11.0).

**(iii) Effect of metal ion cofactors on enzyme activity.** The effect of metal ion cofactors on activity of Ab3 lipase was studied to determine possible enzyme cofactors. The Ab3 lipase activity without any presence of metal ion cofactors at 15°C and pH 7.0 was used to determine the relative activity, as these conditions corresponded to 100% during comparison studies. From Table 2, the presence of Ca^2+^ and Mg^2+^ ions did not provide any significant effect toward the Ab3 lipase activity. Concurrently, the Ab3 lipase activity was reduced up to 30% in the presence of Zn^2+^ and K^+^ ions. The sole presence of Na^+^ ion improved Ab3 lipase activity drastically, up to 30% as the high specific activity of Ab3 lipase was determined, which was 91.5 U/μg ± 0.1. Thus, Na^+^ ion was the most preferrable cofactor for the enhancement of Ab3 lipase activity.

**TABLE 2:** Effects of various metal ions toward the activity of Ab3 lipase^*a*^

Metal ion	Sp act (U/μg)	Rel act (%)
Control	70.3 ± 0.3	100 ± 0.6
CaCl_2_	72.8 ± 0.3	103.6 ± 0.6
ZnCl_2_	58.5 ± 0.4	83.1 ± 0.9
MgCl_2_	68.6 ± 0.5	97.5 ± 1.0
KCl	52.2 ± 0.1	74.2 ± 0.3
NaCl	91.5 ± 0.1	130.1 ± 0.2

a Specific activity of Ab3 lipase without any metal ions was used as the control. Results are expressed as means ± standard deviations from the lipase assay performed in triplicates. Metal ion concentrations were all 20 mM.

**FIG 12:**
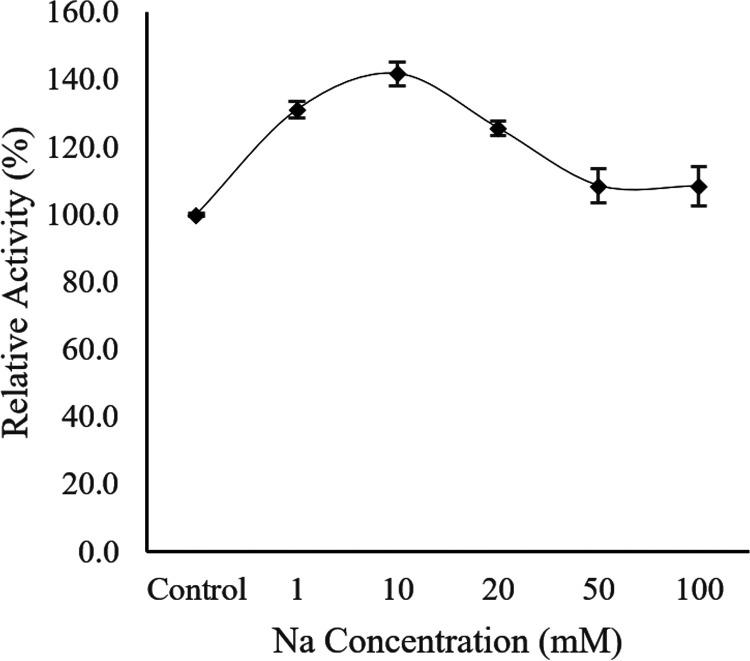
Effect of different Na^+^ ion concentrations toward the activity of Ab3 lipase. Specific activity of Ab3 lipase (72.8 U/μg) without the presence of Na^+^ ion was taken as the control (100%). Results are expressed as means ± standard deviations of the lipase assay performed in triplicates.

Afterwards, the Ab3 lipase activity was further tested in the presence of different Na^+^ ion concentrations of 1 mM, 10 mM, 50 mM, and 100 mM. The potassium phosphate buffer was then used to remove all Na^+^ ions during the lipase assay. The specific activity of Ab3 lipase (72.8 ± 0.4 U/μg) without the presence of Na^+^ ion was used as the control for the relative activity, as it corresponded to 100%. In Fig. 12, Ab3 lipase activity steadily increased when the Na^+^ ion concentration increased toward 10 mM. The maximal specific activity of Ab3 lipase was recorded at 10 mM Na^+^ ion concentration, which was 103.3 ± 3.1 U/μg. By comparison, the Ab3 lipase activity at 10 mM Na^+^ ion concentration increased 10% more than the initial activity shown in Table 2. This further determined that Ab3 lipase preferred to perform in the presence of 10 mM Na^+^ ion to achieve its maximal activity. However, when the concentration of Na^+^ ion was increased to between 20 mM and 50 mM, the enhancement effect was greatly reduced toward the Ab3 lipase activity. The effect was then halted completely at concentrations >50 mM Na^+^ ion, as the Ab3 lipase activity remained unchanged, at 79 ± 3.7 U/μg. This indicated that a high concentration of Na^+^ ion was not required for Ab3 lipase to achieve its maximal activity.

**(iv) Effect of organic solvents on enzyme activity.** The Ab3 lipase activity was studied in the presence of 30% (vol/vol) organic solvent. In Table 3, methanol enhanced Ab3 lipase activity up to 30%, which was 111.3± 0.7 U/μg compared with other organic solvents. No enhancement effects were observed on Ab3 lipase activity in the presence of DMSO and ethanol, as the specific activities of Ab3 lipase remained unaffected, at 88.0 ± 0.3 U/μg and 86.5 ± 0.3 U/μg, respectively. However, the presence of isopropanol heavily affected the Ab3 lipase, with a 25% reduction of its enzymatic performance.

**TABLE 3:** Effects of various organic solvents (30% [vol/vol]) toward the activity of Ab3 lipase^*a*^

Solvent	Sp act (U/μg)	Rel act (%)
Control	86.5 ± 0.9	100 ± 1.3
DMSO	88.0 ± 0.3	101.8 ± 0.5
Methanol	111.3 ± 0.7	128.8 ± 0.8
Ethanol	86.5 ± 0.3	100.0 ± 0.5
Isopropanol	65.4 ± 0.8	75.6 ± 1.5

a Specific activity of Ab3 lipase without any organic solvent was used as the control. Results are expressed as means ± standard deviations of the lipase assay performed in triplicates.

**FIG 13:**
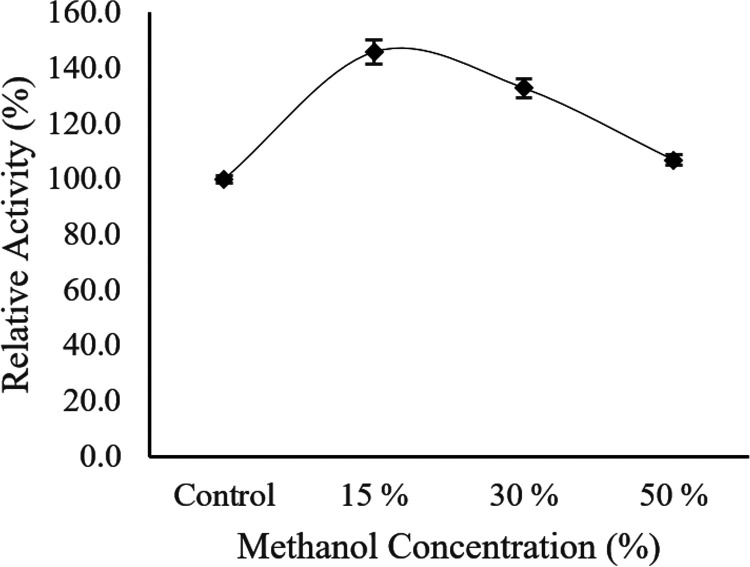
Effect of different methanol concentrations toward the activity of Ab3 lipase. Specific activity of Ab3 lipase (86.5 ± 1.3 U/μg) without the presence of methanol was used as the control (100%). Results are expressed as means ± standard deviations of the lipase assay performed in triplicates.

Afterwards, the Ab3 lipase activity was tested in the presence of different methanol concentrations of 15%, 30%, and 50% (vol/vol). The Ab3 lipase was preincubated in the presence of different methanol concentrations for 30 min before a standard lipase assay. The specific activity of Ab3 lipase (86.5 ± 1.3 U/μg) without the presence of methanol was used as the control for the residual activity, set to correspond to 100%. As shown in Fig. 13, Ab3 lipase activity increased and reached its maximal activity (122± 4.4 U/μg ) in the presence of a 15% methanol concentration. As the concentration of methanol increased between 30% and 50% during the exposure, the enhanced effect was greatly reduced toward the specific activity of Ab3 lipase. The effect was later observed to be halted at a 50% methanol concentration, as the specific activity of Ab3 lipase was 92.3 ± 1.4 U/μg, which was close to the control activity (86.5 ± 1.3 U/μg). Thus, this indicated that Ab3 lipase preferred to perform greatly in the presence of a 15% methanol concentration.

**(v) Effect of detergents on enzyme activity.** The Ab3 lipase activity was studied in the presence of 0.1% (vol/vol) detergents. In Table 4, the presence of SDS heavily decreased the enzymatic performance of Ab3 lipase, up to a 50% loss of its activity, at only 47.0 U/μg. Apart from SDS, cationic detergents such as Tween 20, Tween 80, and TritonX-100 were observed to enhance the Ab3 lipase activity. The lipase activity was enhanced by 67.3% using Tween 20, 41.2% using Tween 80, and 20.9% using Triton X-100. However, Ab3 lipase was observed to function greatly in the presence of Tween 20 compared with other detergents, whereby the specific activity was 144.6 ± 0.5 U/μg.

**TABLE 4:** Effect of various detergents (0.1%) on Ab3 lipase activity^*a*^

Detergent	Sp act (U/μg)	Rel act (%)
Control	86.5 ± 0.9	100 ± 1.3
Tween 20	144.6 ± 0.5	167.3 ± 0.4
Tween 80	122.1 ± 1.1	141.2 ± 1.1
Triton X-100	104.6 ± 1.6	120.9 ± 1.9
SDS	47.0 ± 0.0	54.5 ± 0.0

a Specific activity of Ab3 lipase without any detergent was used as the control. Results are expressed as means ± standard deviations of the lipase assay performed in triplicates.

**FIG 14:**
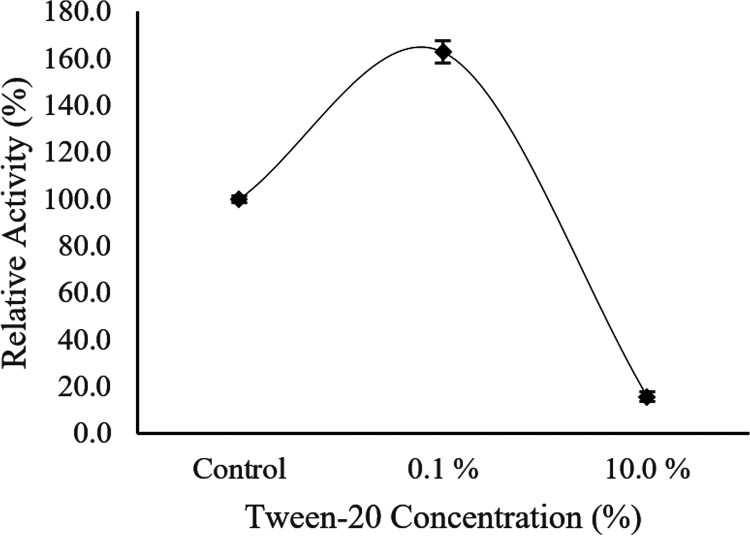
Effect of different Tween 20 concentrations toward the activity of Ab3 lipase. Specific activity of Ab3 lipase (86.5 ± 1.3 U/μg) without the presence of Tween 20 was used as the control (100%). Results are expressed as means ± standard deviations of the lipase assay performed in triplicates.

Afterwards, the Ab3 lipase activity was further tested in the presence of different Tween 20 concentrations, such as 0.1% and 10% (vol/vol). The specific activity of Ab3 lipase (86.5 ± 1.3 U/μg) without the presence of methanol was used as the control for the relative activity, set to correspond to 100%. As shown in Fig. 14, Ab3 lipase performed better with exposure to 0.1% (vol/vol) Tween 20, whereby the specific activity was 140.8 ± 5.0 U/μg. As the concentration of Tween 20 was increased to 10%, the enhanced effect was greatly reduced toward the specific activity of Ab3 lipase. In the presence of 10% Tween 20 concentration, the Ab3 lipase suffered nearly 85% loss in its enzymatic performance, with specific activity at only 13.6 ± 0.2 U/μg. This indicated that Ab3 lipase preferred to function properly at a Tween 20 concentration ≤0.1%.

Comparison of Table 4 and Fig. 14 shows that the Ab3 lipase was much more active in the presence of cationic detergents such as Tween 20, Tween 80, and Triton X-100, as the presence of cationic detergents provided great enhancement effects toward Ab3 lipase activity throughout the study. However, the findings also showed that Ab3 lipase greatly favored the presence of Tween 20 to enhance its enzymatic activity under the circumstance that the concentration was ≤0.1% (vol/vol).

### Kinetic study of *Sphingobacterium* sp. Ab3 lipase.

**FIG 15:**
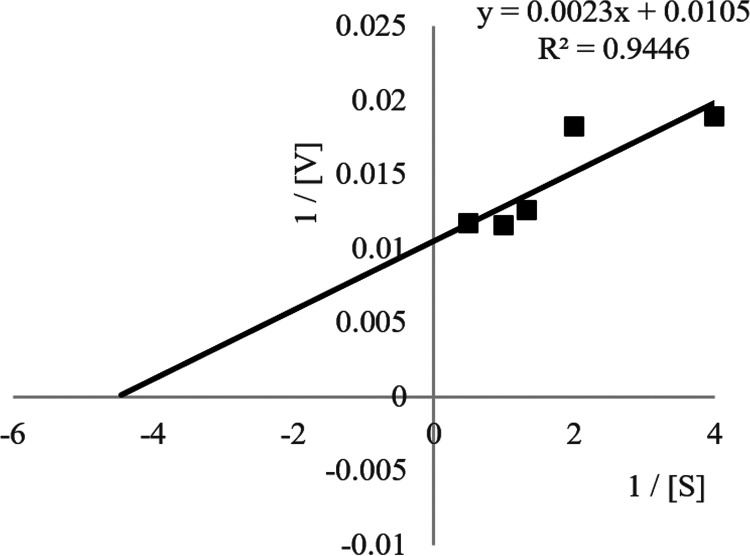
Lineweaver plot of substrate concentrations versus enzyme activity of Ab3 lipase. Results are expressed as means ± standard deviations of the lipase assay performed in triplicates.

The *K_m_*, *V*_max_, and *k*_cat_ values for the Ab3 ligase enzyme were determined using olive oil as substrate. As shown in Fig. 15, the *K_m_*, *V*_max_, and *k*_cat_ values for the Ab3 lipase were 1.6 μM, 95.2 U mL^−1^, and 5.6 × 10^4^ s^−1^. The low *K_m_* and high *k*_cat_ values represented the higher affinity of the enzyme for olive oil, indicating that the enzyme exhibited higher activity against olive oil.

### Features of *Sphingobacterium* sp. Ab3 lipase.

*Sphingobacterium* sp. Ab3 lipase possessed high activities at lower temperatures while retaining its highest stability during lipolytic activities. In addition, *Sphingobacterium* sp. Ab3 lipase preferred a relatively low temperature, 15°C, compared to other cold-adapted lipases shown in Table 5. Most cold-adapted lipases work best at 25°C or 30°C, and in some cases, the cold-adapted lipases from Pseudomonas fluorescens AKM-L5 (63), Candida albicans (64), and *Actinobacter* sp. (65) have been reported to work at lower temperatures, i.e., 10°C and 15°C. Despite having its highest stability at 15°C, *Sphingobacterium* sp. Ab3 lipase retained 80% of its activity at 20°C, demonstrating its moderate thermostability, unlike other bacterial strains shown in Table 5.

**TABLE 5:** Biochemical properties of cold-adapted lipases from different bacteria

Species	Strain	Optimal pH and temp	Unique properties	Reference
*Sphingobacterium* sp.	Ab3	pH 7.0, 15°C	Enhanced activity in presence of Na^+^ ion, cationic detergents, or methanol; high tolerance towards extreme pH environments and presence of DMSO and ethanol	This study
Pseudomonas cepacia	VITCLP4	pH 8.0, 25°C	Alkaline; highly thermostable; high organic solvent tolerance	93
Pseudomonas fluorescens	AKM-L5	pH 7.0, 10°C	Alkaline; high activity at low temp; high organic solvent and metal ion tolerance; high oil degradation activity	63
Pseudomonas fluorescens	KE38	pH 8.5, 25°C	Alkaline; high activity in various organic solvents; enhanced activity in ethanol and acetonitrile	94
Pseudomonas fluorescens	TK-3	pH 8.0, 20°C–30°C	High organic solvent and metal ion tolerance; Ca^2+^ dependent; high enhanced activity by SDS	95
Pseudomonas fluorescens	AMS8	pH 10.0, 30°C	High activity in broad pH range; high organic solvent tolerance	67
Pseudomonas stutzeri	PS59	pH 8.5, 20°C	High stability and activity in broad pH and temp range; high organic solvent tolerance	96
Acinetobacter sp.	XMZ-26	pH 10.0, 15°C	Alkaline; high activity in broad pH range; high enhanced activity by methanol, propanediol, polyethylene glycol 600, and DMSO	97
Burkholderia anthina	NT15	pH 9.5, 30°C	Alkaline; high activity at low temp and the presence of Na^+^ and Mg^2+^ ions	67
Candida albicans		pH 5.0–6.0, 15°C–25°C	High activity at low temp and broad pH range; enhanced activity by Zn^2+^ ion	64
*Candida zeylanoides*	YM-7	pH 7.5–8.0, 28°C	High activity at low temp and broad pH range; enhanced activity by ethanol	98
Psychrobacter cryohalolentis	K5^T^	pH 8.0, 25°C	High activity at low temp and broad pH range; high tolerance against DMSO	69
*Psychrobacter* sp.	C18	pH 8.0, 30°C	High activity at broad pH range; High enhanced activity by various organic solvents and non-ionic detergents.	75

Most cold-adapted lipases from other bacterial species, except Candida albicans (64), were reported to be alkaline enzymes. *Sphingobacterium* sp. Ab3 lipase in this study preferred to function at a similar but wider pH range compared with Candida albicans (64). However, *Sphingobacterium* sp. Ab3 lipase showed extraordinarily high resistance in extreme pH environments by retaining more than 50% of its activity. This showed the greater flexibility in utilization of *Sphingobacterium* sp. Ab3 lipase for beneficial industrial applications, such as detergent and water waste treatment. The presence of Na^+^ ions could significantly enhance *Sphingobacterium* sp. Ab3 lipase activity. Similar cases of an Na^+^ enhancement effect toward enzymatic activity have also been reported for Pseudomonas fluorescens (66), Burkholderia anthina (67), and *Psychrobacter* sp. (68).

*Sphingobacterium* sp. Ab3 lipase highly utilized methanol to enhance lipolytic activities, unlike other cold-adapted bacteria lipases shown in Table 5. *Sphingobacterium* sp. Ab3 lipase also demonstrated similar tolerance for ethanol and DMSO as that shown for Acinetobacter sp. XMZ-26 (65) and Psychrobacter cryohalolentis (69), but it possessed high lipolytic activity in the presence of methanol which is often used in low-temperature biodiesel production. Like most cold-adapted lipases shown in Table 5, *Sphingobacterium* sp. Ab3 lipase exhibits high lipolytic activity in the presence of noncationic detergents, demonstrating its potential applications in the detergent industry.

### Potential applications of *Sphingobacterium* sp. Ab3 lipase.

Ab3 lipase possessed high lipolytic activities at 15°C and attained its highest stability at a low temperature not more than 15°C. Ab3 lipase activities steadily increased until the temperature reached 15°C. Further temperature elevation only decreased lipase activities, in which its structure was slowly degraded due to continuous heat exposures, which explained the sharp drop in its activity after 20°C. This is one of the common traits of most cold-adapted enzymes (70). As these enzymes were exposed to heat continuously, the weak bonds between their structure were easily broken down, resulting in the loss of enzymatic interfaces. Moreover, Ab3 lipase still retained 80% of lipolytic activities at 20°C, reflecting its properties of being moderately thermolabile. At present, most production of commercial foods often involves enzymes or enzyme-catalyzed reactions. The application of cold enzyme in the food industry is cost-effective, as their catalyzed reactions require low energy and can be deactivated using mild heat input (71).

Ab3 lipase is reported to have its optimal lipase activity at pH 7 and 15°C. Different pHs usually affect the ionic distributions within protein molecules, leading to different enzymatic activities. A protein has its least solubility at its isoelectric point (pI), whereby protein aggregations are highly frequent when the electrostatic forces between protein molecules are at a minimum. However, despite exposing the Ab3 lipase to either an extreme acidic or alkaline environment, the Ab3 lipase interestingly retained 50% of its enzymatic activities. At pH 11, Ab3 lipase was able to retain most of its enzymatic activities, implying that Ab3 lipase may possess high tolerance toward extreme pH environments. Furthermore, Ab3 lipase retained its enzymatic activity for 30 min in a slight alkaline environment (pH 7.5 to pH 9.0) or an acidic environment (pH 3.0 to pH 5.0). Nowadays, most detergent industries require enzymes that could work at low temperature and in an alkaline environment (13, 72). At low temperature, the use of cold enzyme could be of great advantage in the detergent industry because of increased use of synthetic fibers, which cannot tolerate temperatures above 50°C. The Ab3 lipase demonstrated such a high tolerance over a wide range of pHs while maintaining its enzymatic activities. This unusual characteristic of Ab3 lipase may be greatly utilized in its future application in the detergent industry.

Na^+^ ion was determined to be the best enzyme cofactor to improve the lipolytic activities of Ab3 lipase. The sole presence of Na^+^ ion increased the lipase activity of Ab3 lipase by up to 30.1%. The group I alkali metal ion cofactors such as Na^+^ ion often play important roles in stabilizing enzyme catalysis intermediates to provide optimal substrate binding sites (73, 74). The binding of Na^+^ ion induced the optimal enzyme conformation to enhance enzyme activity (73, 74). The pH range for optimal activity of Ab3 lipase was between pH 5.0 and 7.0. This indicated that Ab3 lipase could be potentially used for water treatment. During water treatment processes, metal ions are often used to remove or kill other unnecessary microbes. With addition of Na^+^ ions, this would induce high Ab3 lipolytic activities, which would allow more efficient oil removal from water when applied, leading to improvement of water cleanliness.

Recently, much attention has focused on the principle of biodiesel production, which involves only low-temperature transesterification (75). Metal ion cofactors and organic solvents are mainly used for improving biodiesel production (76–78). Addition of organic solvents such as methanol and ethanol during the biodiesel production aid in lower the freezing point of the materials (75, 79). Low-temperature biodiesel production usually is conducted at 20°C, which indicates that cold enzyme is the most suitable choice for the role, since most common enzymes can only be utilized properly at 37°C.

Like most cold-adapted enzymes, the Ab3 protein would be expected to share similar protein structure with high flexibility but low thermostability, as described by other authors (11, 44, 45). The active site of most cold-adapted enzymes is the most heat labile, but its highly flexible conformation allows high enzymatic activity at low temperatures despite being unstable (80). Organic solvent-tolerant enzymes are considered to maintain their stability in organic solvents via noncovalent stabilizing interactions (81). A lipase from *B. sphaericus* 205y exhibited high relative activities in DMSO and methanol while retaining most of its relative activity in ethanol (82). An erstwhile study suggested that the diffusion of DMSO and ethanol into the active site of lysozyme may alter the active site to be more flexible in the place of water molecules (83). Organic solvent can alter the proteins within the aqueous environment by changing the dielectric constant of buffer and penetrating the protein core (33). Dachuri (81) reported that the cold-adapted lipases PML and LipS undergo conformational changes which lead to high conformational stability and rearrangement of amino acids of the enzymatic surface in the presence of DMSO, ethanol, and methanol (81). As discussed above, the presence of methanol possibly induced the conformational changes toward the active site of Ab3 protein, causing an enhanced enzymatic activity of Ab3 protein due to an increase in its structural flexibility. The Ab3 lipolytic activities were increased by nearly 30% in the presence of methanol only. This showed its potential toward the utilization of low-temperature biodiesel production, as Ab3 lipase is considerably stable at such temperatures and may possibly provide highest activities for best results when Na^+^ ion and methanol are added during the process. Moreover, the presence of DMSO and ethanol did not affect the lipolytic activities of Ab3 lipase, indicating that Ab3 lipase could also be used for industrial processes involving these organic solvents without any interferences. As Ab3 lipase was stable in the presence of methanol and ethanol, which are the most-used solvents in biodiesel production (84), Ab3 lipase could be exploited to be used as a biocatalyst to produce fatty acid methyl esters (FAME) to lower the high production cost associated with the chemical catalyst (85).

Lipase is commonly used for formulations in the detergent industry. In the detergent industry, the ideal enzyme should be stable in alkaline environments and active in the presence of surfactants at low temperature (13). Ab3 lipase was determined as a cold-adapted enzyme which had high tolerance in wide pH ranges. In this study, noncationic detergents such as Tween 20, Tween 80, and Triton X-100 were used to emulsify or dissolve the oil contents. This study was conducted to observe any improvements in the enzymatic activities of Ab3 lipase in the presence of any noncationic detergents. Ab3 lipase activities were shown not to be affected by presence of any noncationic detergents. On the contrary, Ab3 lipase activities were found to be increased drastically. This indicated that Ab3 lipase could be applied together with noncationic detergents for better detergent formulations to remove oil stains from cloth fibers in the detergent industry. Moreover, Ab3 lipase activities were reduced by nearly 55% due to the presence of anionic detergents such as SDS. Anionic detergents such as SDS are well known to degrade the structure of the protein, leading to loss of enzymatic activities (86). However, this also implies some considerations for efficient cleanup of industrial leftovers once the industrial process was completed.

### Conclusion.

In this study, *Sphingobacterium* sp. lipase was successfully amplified using specific primers such as Ab3F and Ab3R and cloned into different vectors such as pGEM-T, pCold-I, and pRSFDuet-1. Coexpression of the lipase gene with LEA peptides was used as an efficient way to improve protein expression and increase protein solubility.

The purified Ab3 lipase from *Sphingobacterium* sp. was fully characterized in order to study its general characteristics in terms of temperature, pH, metal ion cofactors, organic solvents, and detergent. The Ab3 lipase was found to work best at 15°C and neutral pH (7.0). Better activity of Ab3 lipase could be attained through the presence of Na^+^ ion, methanol, and cationic detergents. The Ab3 lipase was discovered as a cold-adapted enzyme with these unique characteristics, which could show some beneficial values toward some industrial applications, such as detergent formulation, cold area bioremediation, and low-temperature biodiesel production. Further extended analysis such as protein structure determination should be considered to better understand its enzymatic activities and the limitations for better industrial utilizations.

## MATERIALS AND METHODS

### Source of microbe.

The bacterial isolate was obtained from an Artic soil sample, ARC4, collected by the Polar Research Group of Universiti Sains Malaysia (USM) (87). The sampling took place in August 2011, on the northern coast of Hornsund, Wedel Jarlsberg Land, West Spitsbergen (^o^00′04″N, 15°33′37″E).

### Temperature studies.

The bacterial isolate was cultivated in Luria-Bertani (LB) agar plates at different temperatures: 15°C, 24°C, and 37°C for 48 h to test whether the isolate fit the definition of obligate psychrophile or facultative psychrotroph (87). Obligate psychrophiles are organisms that prefer temperatures close to 0 for growth (70). The term psychrotrophic refers to “organisms previously known as facultative psychrophiles,” or psychrotolerant, with a maximum temperature above 20°C (70). After 48-h cultivation, the cultures were observed for any presence of bacteria growth.

### Bacterial DNA extraction.

Genomic DNA extractions were conducted using a modified cetyltrimethylammonium bromide (CTAB) method (88). Bacterial culture with a total volume of 50 mL was cultivated in Luria-Bertani (LB) broth at 24°C and 180 rpm for 48 h, followed by centrifugation at 2,580 × *g* for 20 min. Supernatant was discarded and the pellet was resuspended with 4 mL of 2× CTAB buffer, followed by incubation at 37°C for 1 h. The mixture was gently shaken twice every 15 min during incubation at 37°C for 1 h. A total volume of 50 μL of lysozyme (50 mg/mL) was added into the tube, followed by incubation at 37°C for 1 h. Then, 50 μL of 10% SDS was added and incubated at 37°C for 1 h. The process was followed by addition of 2 mL of chloroform:isoamyl (24:1) and a 1/10 volume of 10% CTAB solution, and the tube was then incubated overnight at 37°C and 180 rpm.

The next day, the tube was centrifuged at 2,580 × *g* for 10 min, and only 500 μL of the uppermost layer of the supernatant was transferred into a sterile tube. One volume of phenol:chloroform:isoamyl alcohol (25:24:1) was then added and mixed thoroughly by inverting tubes 20 times. The emulsion produced was centrifuged at 13,523 × *g* for 1 min, and two phases were achieved. The uppermost layers formed were then transferred into new 1.5-mL sterile tubes. The process was then repeated at least twice.

After that, 1 volume of chloroform was added and mixed thoroughly by inverting the tubes 20 times. The emulsion produced was then centrifuged at 13,523 × *g* for 20 min, and two phases were obtained. The uppermost layer was then transferred into new sterile 1.5-mL tubes. The process was then repeated at least twice to remove the remaining phenol.

Two volumes of 95% ethanol were added and mixed thoroughly by inverting until DNA precipitation was formed. After centrifugation at 13,523 × *g* for 20 min, supernatant was discarded, and the pellet was air dried for 30 min to allow the evaporation of the remaining ethanol from the pellet. The dry pellet was dissolved in 30 μL of deionized water and stored at −20°C for long-term storage before being used.

### 16S rDNA gene sequencing.

The acquired genomic DNA was used as DNA template for species identification via 16S rDNA gene sequencing. The 16S rDNA genes from the extracted DNA were generated via PCR using 27F primer (5′-AGA GTT TGA TCC TGG CTC AG-3′) and 1492R primer (5′-GGT TAC CTT GTT ACG ACT T-3′). PCR was set up as shown in Table 6. PCR conditions began with an initial denaturation at 95°C for 5 min, followed up by 29 subsequent cycles of 95°C for 30 s, 58°C for 30 s, 72°C for 1 min, and final extension at 72°C for 5 min. Results of PCR amplification were observed through 1% gel electrophoresis. Successfully verified PCR products were purified through use of a HiYield Plus gel PCR DNA minikit (Real Biotech Corporation, Taiwan) and were sent to MyTACG Bioscience Enterprise for gene sequencing. Analysis of gene sequencing results was performed through alignment of the forward and reverse sequences, followed by further verification toward aligned sequences via NCBI BLAST. Through analysis of 16S rDNA gene sequencing results, the bacterial isolate from the Artic soil sample, ARC4, was identified as *Sphingobacterium* sp. and was 99.0% identical.

**TABLE 6:** PCR setup for 16S rRNA gene sequencing

Ingredient	Vol (μL)
10× *Taq* reaction buffer	5.0
Deoxynucleoside triphosphate (dNTP; 10 mM)	2.0
27F primer (10 μM)	2.0
1492R primer (10 μM)	2.0
DNA template (genome DNA)	2.0
*Taq* DNA polymerase	2.0
Deionized water	35.0
Total vol	50.0

### Screening of the lipase-producing strain.

In this study, the bacterial isolate was cultivated in LB medium at 24°C. A tributyrin test and rhodamine B test were used for detection of *Sphingobacterium* sp. lipase activity. The tributyrin test was used for initial detection of any presence of esterase or lipases, while the rhodamine B test provided further insight for detecting only lipase activity. A single colony of *Sphingobacterium* sp. was isolated and incubated on a 1% tributyrin agar plate and an olive oil-rhodamine B agar plate (1% [vol/vol] olive oil; 0.001% [wt/vol] rhodamine B solution) at 24°C for 48 h.

### PCR amplification.

The specific primers Ab3F (5′-ATG AAC ACA ATA GAT CAG GAG CTT TTA G-3′) and Ab3R (5′-TCA TTT ATC TTG ATT TAC TG-3′) were designed from the region of positions 1036228 to 1037178 within the whole-genome sequence data of *Sphingobacterium* sp. PM2-P1-29 (accession number LK931720) to amplify the *Sphingobacterium* sp. lipase gene. The nucleotide sequence of *Sphingobacterium* sp. PM2-P1-29 contained 951 bp, which encoded 316 amino acids. Figure 1 shows that the estimated molecular weight of lipase gene product was 36 kDa. PCR was set up as shown in Table 7. PCR conditions began with an initial denaturation at 95°C for 5 min, followed by by 29 subsequent cycles of 95°C for 30 s, 58°C for 30 s, 65°C for 30 s, and final extension at 65°C for 5 min. Results of PCR amplification were observed after 1% gel electrophoresis. Successfully verified PCR products were purified through a HiYield Plus gel/PCR DNA minikit (Real Biotech Corporation, Taiwan) and were sent to MyTACG Bioscience Enterprise for gene sequencing. Successful PCR products were then labeled as the Ab3 gene and were purified through use of a PCR purification kit (Real Biotech Corporation, Taiwan). Analysis of Ab3 gene sequencing results was performed and verified through NCBI BLAST for further confirmation.

**TABLE 7:** PCR setup for PCR amplification of the Ab3 gene

Ingredient	Vol (μL)
10× *Taq* reaction buffer	5.0
dNTP (10 mM)	2.0
Ab3F primer (10 μM)	2.0
Ab3R primer (10 μM)	2.0
DNA template (genome DNA)	2.0
*Taq* DNA polymerase	2.0
Deionized water	35.0
Total vol	50.0

### Restriction digestion setup for the plasmid and lipase genes.

The restriction digestion setups were performed by using 10× NEB CutSmart buffer, specific restriction enzymes, and deionized water (http://international.neb.com/protocols/2012/12/07/optimizing-restriction-endonuclease-reactions). The DNA samples used were either specific plasmids (pCold-I and pRSFDuet-1) or PCR products. The concentrations of specific plasmids or PCR products were estimated using a Nanodrop spectrophotometer. Using data obtained, the concentrations of specific plasmids or PCR products were added accordingly to complete the reaction setup as shown in Table 8. Each restriction digestion reaction mixture was prepared in a new 200-μL sterile tube and mixed thoroughly, followed by incubation at 37°C for 30 min. The incubation duration extend to 1 h or longer if restriction digestion reactions were suspected to be slow.

**TABLE 8:** Standard restriction digestion reaction setup

Ingredient	Amt
10× NEB CutSmart buffer	5 μL (1×)
DNA	1 μg
Restriction enzyme	10 U
Deionized water	For 50-μL
Total vol	50 μL

### Ligation reaction setup for the lipase gene.

The ligation reaction setups were performed by using 5× ligation buffer, specific plasmids (pCold-I and pRSFDuet-1), insert DNA (purified and digested PCR products), T4 DNA ligase, and deionized water. The concentrations of specific plasmids and insert DNA were estimated using a Nanodrop spectrophotometer. The data obtained were used to calculate the volumes needed for specific plasmids and PCR products to set up the ligation reaction using an *in silico* ligation calculator (http://www.insilico.uni-duesseldorf.de/Lig_Input.html). The ligation ratio of plasmid and insert DNA used was 1:3. Each ligation reaction mixture was prepared in new sterile 1.5-mL tubes and was mixed gently through pipetting, followed by overnight incubation at 4°C for maximum number of transformants.

### Preparation of E. coli competent cells.

The E. coli competent cells [strains JM109 and BL21(DE3)] were prepared using the calcium chloride method (89, 90). A single colony of E. coli cells was inoculated into 50 mL LB medium, followed by overnight incubation at 37°C and 180 rpm. The culture with a total volume of 500 μL was inoculated into 50 mL LB broth and was incubated at 37°C and 180 rpm for 2 h, until the optical density at 715 nm (OD_715_) reached 0.4. The culture was then transferred into a new sterile 50-mL tube and was centrifuged at 1,893 × *g* and 4°C for 7 min. After discarding supernatant, the pellet was resuspended gently with 10 mL ice-cold CaCl_2_ solution (60 mM CaCl_2_, 20% glycerol), followed by centrifugation at 2,580 × *g* and 4°C for 5 min. Supernatant was discarded, and the pellet was gently resuspended with 2 mL ice-cold CaCl_2_ solution. The tube containing resuspended cells was kept on ice for 30 min, followed by centrifugation at 2,580 × *g* and 4°C for 5 min. The pellet was resuspended gently with 2 mL ice-cold CaCl_2_ solution, and 200 μL of resuspended cells was then transferred to new 1.5-mL sterile tubes. The tubes were then kept at −80°C for any future cell transformation usages.

### Cloning of lipase gene via pGEM-T plasmid.

The restriction sites BamHI and EcoRI were introduced on either side of the gene encoding the Ab3 lipase by PCR using the following pair of primers: Ab3G-F primer (5′-CAA TTC GGA TCC ATG AAC ACA ATA GAT CAG GAG CTT TTA G-3′) and Ab3G-R primer (5′-GCC GCC GAA TTC TCA TTT ATC TTG ATT TAC TG-3′); the underlined bases within the primers refer to the addition of restriction sites. PCR was set up similarly (Table 6) using modified Ab3 lipase gene as the sample. PCR conditions began with an initial denaturation at 95°C for 5 min, followed up by 29 subsequent cycles of 95°C for 30 s, 58°C for 30 s, 65°C for 30 s, and final extension at 65°C for 5 min. The PCR products were purified through a HiYield Plus gel PCR DNA minikit (Real Biotech Corporation, Taiwan), followed by with restriction digestions.

Digested Ab3 lipase gene was purified using a HiYield Plus gel PCR DNA minikit (Real Biotech Corporation, Taiwan) and ligated with pGEM-T plasmid after an overnight incubation at 4°C. The ligated products were mixed with E. coli JM109, followed by heat shock treatment at 42°C in a water bath for 50 s. LB broth (600 μL) was added into the tube and mixed thoroughly, followed by incubation at 37°C and 180 rpm for 45 min. Finally, the tubes containing transformants were centrifuged at 13,523 × *g* for 1 min. Supernatant was removed and the pellet was resuspended using 60 μL of fresh LB broth. Resuspended cells were placed onto each prepared LB–ampicillin–IPTG–5-bromo-4-chloro-3-indolyl-β-d-galactopyranoside plate, followed by overnight incubation at 37°C.

The positive transformants were verified through colony PCR. The colony PCR was set up as shown in Table 9. The colony PCR conditions began with an initial denaturation at 95°C for 5 min, followed up by 29 subsequent cycles of 95°C for 30 s, 58°C for 30 s, 65°C for 30 s, and final extension at 65°C for 5 min. The PCR products from true-positive transformants were then purified and sent to MyTACG Bioscience Enterprise for sequencing to conduct further analysis through NCBI BLAST. The final validated positive transformants were then kept at −80°C as glycerol stocks.

**TABLE 9:** PCR setup for colony PCR toward positive pGEM-T transformants

Ingredient	Vol (μL)
10× *Taq* reaction buffer	5.0
dNTP (10 mM)	2.0
Ab3G-F primer (10 μM)	2.0
Ab3G-R primer (10 μM)	2.0
Positive transformants	1 scrap
*Taq* DNA polymerase	2.0
Deionized water	37.0
Total vol	50.0

### Subcloning of lipase gene via pCold-I plasmid.

The pCold-I plasmid (TaKaRa, Japan) was used as an expression vector for the expression of Ab3 lipase. pCold-I induced the cold shock protein A (*cspA*) promoter for expression of high-purity, high-yield recombinant protein in E. coli at 15°C. The recombinant pGEM-T plasmid containing Ab3 gene was digested with restriction enzymes such as EcoRI and BamHI, purified using gel extraction purification method, and ligated with digested pCold-I plasmid after an overnight incubation at 4°C. The ligated products were mixed with E. coli BL21(DE3), followed by heat shock treatment at 42°C in a water bath for 50 s. LB broth (600 μL) was added into the tube and mixed thoroughly, followed by incubation at 37°C and 180 rpm for 45 min. Finally, the tubes containing transformants were centrifuged at 13,523 × *g* for 1 min. Supernatant was removed and the pellet was resuspended using fresh LB broth (60 μL). Resuspended cells were placed onto LB medium containing ampicillin (100 mg/mL), followed by overnight incubation at 37°C.

The positive transformants were verified through colony PCR. The colony PCR was set up similarly as shown in Table 9. Colony PCR conditions began with an initial denaturation at 95°C for 5 min, followed by 29 subsequent cycles of 95°C for 30 s, 58°C for 30 s, 65°C for 30 s, and final extension at 65°C for 5 min. The PCR products from true-positive pCold-I transformants were then purified using a HiYield Plus gel PCR DNA minikit and sent to MyTACG Bioscience Enterprise for sequencing to conduct further analysis through NCBI BLAST. The final validated positive pCold-I transformants were then kept at −80°C as glycerol stocks.

### Expression of recombinant protein via pCold-I plasmid.

Transformed E. coli BL21(DE3) cells containing the pCold-I plasmid for expression of the target protein were cultured in a conical flask containing 50 mL of LB medium with ampicillin (100 mg/mL) at 37°C and 180 rpm for 2 h. When the OD_715_ reached 0.5, the culture was cooled at 15°C for 30 min and IPTG was added as an inducer to the culture medium at a final concentration of 0.1 mM. After induction, the cultivation was continued at 15°C and 180 rpm. Protein expression levels at 4, 8, 12 and 24 h were observed by collecting 1 mL culture at each time interval during ongoing incubation for further analysis through SDS-PAGE. The cultivated microorganism was harvested by centrifugation at 1,893 × *g* for 6 min and suspended in 0.1 M sodium phosphate buffer (pH 7.4).

### Introduction of LEA peptides in protein expression.

**(i) Plasmid construction for Ab3 and LEA peptide gene.** The pRSFDuet-1 plasmid (Novagen Merck KGaA, Darmstadt, Germany) was used as an expression vector for the coexpression of Ab3 lipase and LEA peptide. pRSFDuet-1 contains 2 multiple-cloning sites, namely, MCS-1 and MCS-2, together with ribosome-binding sites and a T7 *lac* promoter vector. The latter recombinant pRSFDuet-1 genes are often equipped with an N-terminal His-tag marker to allow simple purification after protein expression. MCS-1 and MCS-2 cloning sites were subcloned with Ab3 lipase and LEA genes, respectively. At the cloning site of MCS-2, LEA-I and LEA-K peptide genes were subcloned into the expression vector as described previously. The gene encoding LEA-I and the LEA-K peptide gene were subcloned into the pRSFDuet-1 vector digested by the restriction enzymes EcoRV and XhoI.

The following oligo-DNA pairs encoded these LEA peptides: LEA-I (5′-GAT ATA CAT ATG GAT GCG AAA GAC GGG ACG-3′ and 5′-CTT TCG TCC CGT CTT TCG CAT CCA TAT GTA TAT C-3′) and LEA-K (5′-GCG AAA GAC AAA ACG AAA GAA GCA AAA GAA TAA C-3′ and 5′-TCG AGT TAT TCT TTT GCT TTT TCT TTC GTT TTG TCT TTC GC-3′).

The oligo-DNAs were phosphorylated at 37°C for 1 h by using T4 polynucleotide kinase (New England Biolabs), followed by treatment at 65°C for 20 min. Each pair was then hybridized in Tris-HCl buffer (pH 8.0) containing 50 mM KCl. The hybridized DNA fragments were ligated with the vector digested by the restriction enzymes EcoRV and XhoI. The construction of the expression vector was verified by DNA sequencing using FASMAC (Atsugi, Japan). All the oligo-DNAs were obtained from GeneNet (Fukuoka, Japan).

The restriction sites BamHI and HindIII were introduced on either side of the gene encoding the Ab3 lipase by PCR using the following pair of primers: Fw primer (5′-CCC CCC CCG GAT CCG ATG AAC ACA ATA-3′) and Rev primer (5′-CCC CCC AAG CTT TCA TTT ATC TTG ATT-3′); the underlined portions indicate the addition of restriction sites. The amplified DNA fragment was digested with BamHI and HindIII, purified by agarose gel electrophoresis, and ligated into the digested pRSFDuet-1 vector. The Ab3 lipase gene was subcloned into the MCS-1 cloning site of the digested pRSFDuet-1 vector in the same way as described previously from the subcloning in pCold-I vector. The transformants were cultivated on LB medium containing kanamycin (100 mg/mL) overnight at 37°C. The verification of positive transformants was performed using colony PCR as described in Table 9. Colony PCR conditions began with an initial denaturation at 95°C for 5 min, followed by 29 subsequent cycles of 95°C for 30 s, 58°C for 30 s, 65°C for 30 s, and final extension at 65°C for 5 min. The final validated positive transformants were then purified using a HiYield Plus gel PCR DNA minikit and sent to MyTACG Bioscience Enterprise for sequencing to conduct further analysis through NCBI BLAST. Finally, final validated positive transformants were kept at −80°C as glycerol stock.

**(ii) Coexpression of recombinant protein and LEA peptides.** The transformed E. coli BL21(DE3) cells containing the pRSFDuet-1 plasmid for coexpression of LEA peptide and target protein were cultured in a conical flask containing 50 mL of LB medium with kanamycin (100 mg/mL) at 37°C and 180 rpm for 2 h. When the OD_715_ reached 0.5, the culture was cooled at 15°C for 30 min and IPTG was added as an inducer to the culture medium at a final concentration of 0.1 mM. After induction, the cultivation was continued at 24°C and 180 rpm. Protein expression levels at 4, 8, 12, and 24 h were observed by collecting 1 mL of culture at each time interval during ongoing incubation for further analysis through SDS-PAGE. The cultivated microorganisms were harvested by centrifugation at 1,893 × *g* for 6 min and suspended in 0.1 M sodium phosphate buffer (pH 7.4).

**(iii) Purification of expressed protein.** Before purification, the cell lysis was conducted using an ultrasonic disruptor UD-201 (Tomy Seiko Co., Ltd, Japan). A total volume of 50 mL harvested culture in a sterilized centrifuge tube was extensively sonicated with 10 to 20 intermittent pulses (pulse, 0.6 s; interval, 0.4 s; output level, 5) in an ice bath with 5-s work time and 10-s rest time in the repeated cycle of 30 until the solution became translucent. After centrifugation with 13,523 × *g* for 6 min, supernatant (soluble protein) and pellet (insoluble protein) were collected. Purification of expressed protein was then conducted using HisTalon metal chromatography (TaKaRa, Japan). A 200-μL aliquot of metal resin was first transferred into a sterile His-tagged column containing a glass filter with a pore size of 10 to 20 μm, followed by washing using 100 mM phosphate buffer (pH 7) for removal of ethanol before enzyme purification. Two milliliters of supernatant was transferred into the column, and 1 mL of flowthrough was collected as a control for SDS-PAGE analysis. The expressed proteins trapped within the column were then eluted by adding 2 mL of phosphate buffer containing 10 mM imidazole. Two tubes containing 500 μL of flowthrough were collected for SDS-PAGE analysis during the process. Elution was again repeated by using phosphate buffers containing imidazole at different concentrations, such as 20 mM, 40 mM, and 60 mM. The results were then observed through SDS-PAGE. Successful purified proteins were then labeled as Ab3 lipase.

**(iv) Estimation of protein concentration.** Estimation of protein concentration was conducted using a Bradford assay (91). The concentration of purified Ab3 lipase was estimated at 595 nm based on the standard curve of for BSA (0 to 100 μg/mL) in sodium phosphate buffer (pH 7.0). Duplicate aliquots of 0.5 mg/mL BSA (5, 10, 15, and 20 μL) were placed into eight sterile 1.5-mL tubes, and each was diluted to 100 μL with 0.1 M sodium phosphate buffer (pH 7.0). Meanwhile, 100 μL of 0.1 M sodium phosphate buffer (pH 7.0) was placed into two more sterile 1.5-mL tubes which served as the blank tubes. A 1-mL volume of Coomassie brilliant blue solution (1×) was added into the tubes and mixed thoroughly by inverting several times. The tubes were then allowed to stand for 10 min at room temperature (24°C). Absorbances of each tube were measured through a spectrophotometer at 595 nm to plot the graph of absorbance at 595 nm versus protein concentration. The unknown concentration of purified Ab3 lipase was estimated at 595 nm based on the standard curve for BSA (0 to 100 μg/mL) in 0.1 M sodium phosphate buffer (pH 7.0). After estimation of purified Ab3 lipase concentration, the protein concentration used for latter biochemical characterizations was then adjusted to 1.0 μg/mL.

**(v) Lipase assay.** The lipase assay was conducted using a colorimetric method with olive oil as substrate (92). The reaction mixture contained 1 mL of purified Ab3 lipase (1.0 μg/mL), 2.5 mL of olive oil emulsion (a mixture of olive oil and 0.1 M sodium phosphate buffer [pH 7.0] in a 1:1 ratio with 1% polyvinyl alcohol [PVA] added) and 20 μL of 20 mM calcium chloride was added and laterally shaken at 15°C and 180 rpm for 30 min. The enzyme reaction in the emulsion system was stopped by adding 1 mL of 6 M HCl and 5 mL of isooctane, followed by vortexing for 1 min. A 4-mL upper isooctane layer containing the free fatty acid was transferred to a test tube and properly mixed with 1 mL copper reagent [prepared by adjusting the solution of 5% (wt/vol) copper(II) acetate-1-hydrate to pH 6.1 with pyridine], followed by incubation at room temperature on the lab bench for 30 min. Free fatty acid dissolved in isooctane was determined by measuring the absorbance of the upper layer at 715 nm after mixture settlement. Lipase activity was determined by measuring the amount of free fatty acid released based on the standard curve of oleic acid (0 to 2,000 μmole) in isooctane. One unit of lipase activity was defined as 1.0 μmol of free fatty acid liberated min^−1^ and reported in units per milliliter. The lipase assay was performed in triplicates, and the data results are expressed as mean ± standard deviations. Lipase activity (in units per milliliter) was calculated using the following equation: lipase activity = amount of free fatty acid released (in micromoles)/time (in minutes). Specific activity of the enzyme (in units per microgram) was determined as follows: specific activity = lipase activity (in units per milliliter)/estimated protein concentration of enzyme (in micrograms per milliliter).

### Biochemical characterization.

**(i) Effect of temperature on enzyme activity and thermostability.** For enzyme activity, purified Ab3 lipase was tested in a standard lipase assay protocol by incubating in sodium phosphate buffer (0.1 M, pH 7.0) at different temperatures ranging from 4°C to 50°C. For enzyme stability, purified Ab3 lipase was first preincubated at different temperatures for 30 min, followed by a standard lipase assay protocol. Residual activity of the enzyme was studied afterwards.

**(ii) Effect of pH on enzyme activity and stability.** For enzyme activity, purified Ab3 lipase was tested with a standard lipase assay protocol by incubating with 2.5 mL of olive oil emulsion at different pHs ranging from 3 to 11 in different buffers. The buffers used were sodium acetate buffer (0.1 M, pH 3.0 to 5.5), sodium phosphate buffer (0.1 M, pH 6.0 to 7.5), Tris-HCl buffer (0.1 M, pH 8.0 to 9.0), and glycine-NaOH buffer (0.1 M, pH 9.5 to 12.0). For enzyme stability, purified Ab3 lipase was first preincubated at different pHs for 30 min, followed by a standard lipase assay protocol. Residual activity of the enzyme was later determined.

**(iii) Effect of metal ion cofactors on enzyme activity.** The metal ion cofactors assay was conducted at 15°C and 200 rpm for 30 min by treating the purified Ab3 lipase (1.0 μg/mL) with 200 μL of a 20 mM concentration of different salts, including CaCl_2_, ZnCl_2_, KCl, MgCl_2_, and NaCl in sodium phosphate buffer (0.1 M, pH 7.0).

**(iv) Effect of organic solvent on enzyme activity.** For enzyme stability, purified Ab3 lipase (1.0 μg/mL) was first preincubated with 30% (vol/vol) organic solvent sodium phosphate buffer (0.1 M, pH 7.0) at 15°C for 30 min, followed by a standard lipase assay protocol. The selected organic solvents were DMSO, methanol, ethanol, and isopropanol.

**(v) Effect of detergent on enzyme activity.** For enzyme stability, purified Ab3 lipase (1.0 μg/mL) was first preincubated with 0.1% (vol/vol) detergent in sodium phosphate buffer (0.1 M, pH 7.0) at 15°C for 30 min, followed by a standard lipase assay protocol. The selected detergents were Tween 20, Tween 80, Triton X-100, and SDS.

### Kinetic study of *Sphingobacterium* sp. Ab3 lipase.

The *K_m_*, *V*_max_, and *k*_cat_ values of the enzyme were determined under standard assay conditions using 0.25 to 2% (vol/vol) olive oil as substrate. The lipase assay was performed in triplicates, and the data results are expressed as means ± standard deviations. The constant values were calculated by fitting data to a linear regression using a Lineweaver-Burk plot.
